# Distribution of HCV genotypes in Belgium from 2008 to 2015

**DOI:** 10.1371/journal.pone.0207584

**Published:** 2018-12-05

**Authors:** Lobna Bouacida, Vanessa Suin, Veronik Hutse, Michaël Boudewijns, Reinoud Cartuyvels, Laurent Debaisieux, Emmanuel De Laere, Marie Hallin, Nicolas Hougardy, Katrien Lagrou, Els Oris, Elizaveta Padalko, Marijke Reynders, Gatien Roussel, Jean-Marc Senterre, Michel Stalpaert, Dominique Ursi, Carl Vael, Dolores Vaira, Jos Van Acker, Walter Verstrepen, Steven Van Gucht, Benoit Kabamba, Sophie Quoilin, Gaëtan Muyldermans

**Affiliations:** 1 Sciensano, Laboratory of medical microbiology, Brussels, Belgium; 2 Sciensano, Viral diseases, Brussels, Belgium; 3 National reference center for hepatitis viruses, Belgium, Belgium; 4 AZ Groeninge, Campus Kennedylaan Laboratorium, Kortrijk, Belgium; 5 Jessa Ziekenhuis, Klinisch Laboratorium, Hasselt, Belgium; 6 LHUB-ULB site Anderlecht, CUB-Hôpital Erasme, Brussels, Belgium; 7 AZ Delta, Laboratorium, Roeselare, Belgium; 8 LHUB-ULB, Site Porte De Hal, Brussels, Belgium; 9 Clin. Sud Luxembourg, Site St-Joseph Labo D'analyses Médicales, Arlon, Belgium; 10 UZ Leuven, Clinical Department of Laboratory Medicine, Leuven, and-KU Leuven, Department of Microbiology and Immunology, Leuven, Belgium; 11 Ziekenhuis Oost-Limburg, Labo Klinische Biologie, Genk, Belgium; 12 UZ Ghent, Clinical Biology–Medical Microbiology Laboratory, Ghent, Belgium; 13 AZ Sint-Jan Brugge-Oostende AV, Laboratory Medicine, Brugge, Belgium; 14 Clinique St. Pierre, Laboratoire De Biologie Clinique, Ottignies, Belgium; 15 Ch Regional De La Citadelle, Laboratoire, Liège, Belgium; 16 Algemeen Medisch Labo, Antwerp, Belgium; 17 University Hospital Antwerp, Laboratory of Molecular Diagnostics in Microbiology, Antwerp, Belgium; 18 AZ KLINA, Clinical Laboratory, Brasschaat, Belgium; 19 CHU de Liège, Laboratoire de Référence SIDA-ULg, Liège, Belgium; 20 AZ Sint Lucas, Laboratorium, Gent, Belgium; 21 ZNA, Klinisch Laboratorium Campus Middelheim, Antwerp, Belgium; 22 Cliniques Universitaires Saint-Luc, Labo Biologie Clinique Ria, Brussels, Belgium; 23 Sciensano, Epidemiology of infectious diseases, Brussels, Belgium; University of Cincinnati College of Medicine, UNITED STATES

## Abstract

**Background:**

The knowledge of circulating HCV genotypes and subtypes in a country is crucial to guide antiviral therapy and to understand local epidemiology. Studies investigating circulating HCV genotypes and their trends have been conducted in Belgium. However they are outdated, lack nationwide representativeness or were not conducted in the general population.

**Methods:**

In order to determine the distribution of different circulating HCV genotypes in Belgium, we conducted a multicentre study with all the 19 Belgian laboratories performing reimbursed HCV genotyping assays. Available genotype and subtype data were collected for the period from 2008 till 2015. Furthermore, a limited number of other variables were collected: some demographic characteristics from the patients and the laboratory technique used for the determination of the HCV genotype.

**Results:**

For the study period, 11,033 unique records collected by the participating laboratories were used for further investigation.

HCV genotype 1 was the most prevalent (53.6%) genotype in Belgium, with G1a and G1b representing 19.7% and 31.6%, respectively. Genotype 3 was the next most prevalent (22.0%). Further, genotype 4, 2, and 5 were responsible for respectively 16.1%, 6.2%, and 1.9% of HCV infections. Genotype 6 and 7 comprise the remaining <1%. Throughout the years, a stable distribution was observed for most genotypes. Only for genotype 5, a decrease as a function of the year of analysis was observed, with respectively 3.6% for 2008, 2.3% for 2009 and 1.6% for the remaining years.

The overall M:F ratio was 1.59 and was mainly driven by the high M:F ratio of 3.03 for patients infected with genotype 3. Patients infected with genotype 3 are also younger (mean age 41.7 years) than patients infected with other genotypes (mean age above 50 years for all genotypes). The patients for whom a genotyping assay was performed in 2008 were younger than those from 2015.

Geographical distribution demonstrates that an important number of genotyped HCV patients live outside the Belgian metropolitan cities.

**Conclusion:**

This national monitoring study allowed a clear and objective view of the circulating HCV genotypes in Belgium and will help health authorities in the establishment of cost effectiveness determinations before implementation of new treatment strategies.

This baseline characterization of the circulating genotypes is indispensable for a continuous surveillance, especially for the investigation of the possible impact of migration from endemic regions and prior to the increasing use of highly potent direct-acting antiviral (DAA) agents.

## Introduction

The hepatitis C virus (HCV) belongs to the genus *Hepacivirus*, https://en.wikipedia.org/wiki/Hepacivirusa member of the family *Flaviviridae* and is a major cause of chronic liver disease, liver cirrhosis and hepatocellular carcinoma. It is a globally prevalent pathogen and a leading cause of morbidity and mortality [1]. The infection is predominantly acquired by blood-borne transmission of the virus besides a low risk of sexual transmission. The spread during the decades prior to the discovery of HCV was probably caused by contaminated blood, blood products, injecting drug use and other routes.

An action plan for the health sector response to viral hepatitis, including HCV, in the WHO European region was published and aims to eliminate viral hepatitis by 2030 through the reduction of transmission, morbidity and mortality and by ensuring equitable access to comprehensive prevention, testing, care and treatment service for all [2]. In order to achieve these goals, the current number of HCV cases (n = 70,000) as of 2011 in Belgium, number of cases diagnosed (n = 22,900) with approximately 2900 new viremic HCV diagnoses annually, and the number of patients treated (n = 710/yearly) were estimated based on literature review in combination with expert panel discussions [3]. Currently no screening strategy is installed in Belgium. However almost 750.000 persons, nearly 7% of the Belgian population) are yearly screened for the presence of HCV antibodies, a test reimbursed by the social security. [4].Also the confirmation test, based on molecular diagnosis either by qualitative or quantitative analysis and the genotyping are reimbursed by the social security system in Belgium. The last one is mainly performed when intention to treat.

Given its wide genetic variability, HCV classification by sequence analysis was recently updated and comprises 7 genotypes (1–7) and 67 subtypes [5]. A Web resource hosted by the International Committee for Taxonomy of Viruses (ICTV) maintains and regularly updates tables of reference isolates, accession numbers and annotated alignments [6].

Genotype 1, specifically subtypes 1a and 1b are the most prevalent in Europe and USA. Nevertheless, there is a growing significance of genotype 3 being the second most common genotype reported in all European countries except for Italy and Romania [7]. Substantial regional differences appear to exist in the distribution of HCV genotypes [8, 9]. The subtypes 1a, 1b, 2a, and 3a are widely distributed across the globe and account for a large proportion of HCV infections in high income countries. Other subtypes are more restricted to specific regions/continents i.e. genotype 4 in Central Africa and the Middle East, genotype 5 in Southern Africa and 6 in South East Asia. Genotype 7 has been recently isolated from central African immigrants in Canada [10].

Major improvements in antiviral therapies for HCV have been made during the last decades. New short-course oral treatments can achieve cure in most patients, including those previously considered difficult to treat, although long-term follow-up data are not yet available [11, 12]. These advances are set to be important components of a new global health strategy [13]. The clinical relevance of HCV genotyping has been demonstrated in the day-to-day clinical management of HCV patients. Previously, genotyping was clinically important in predicting potential response to interferon-based therapy and in determining the required duration of such therapy. Currently, with the advent of the expensive direct acting antivirals (DAA), genotyping becomes rather a tool to choose for the most economically favorable therapy choice. Therefore, the development of national treatment strategies using DAA therapies requires a detailed knowledge of the circulating HCV genotypes and subtypes. Also the accuracy of the cost benefit analysis can profit from the availability of this genotyping information.

The sensitivity of detection of HCV RNA with qualitative and quantitative HCV techniques may vary according to the choice of primers and amplification region [14]. Similarly the used primers and target amplification region might influence the accuracy of the genotyping result. Also, the accuracy of the genotyping result is of utmost importance for the success of the patient personalized therapy [15].

At present, the duration of treatments and their success rates remain dependent on the HCV genotype and subtype. At the level of HCV diagnostic assays, genotype dependent differences had major implications for the use of HCV diagnostic tests especially in geographic areas with a high prevalence of HCV genotypes that are phylogenetically distant from genotype 1a, the prototype sequence used in the development of these commercial diagnostic assays in previous years [16].

Moreover, HCV genotyping is a relevant epidemiological tool in outbreak and transmission investigation for tracing the source [17, 18,19]. Recently, the impact of migration flows in Europe on the spread of infectious diseases has been demonstrated [20] and genotyping will be the most appropriate tool to monitor the effect of migration flows on HCV dynamics.

Concerning the development of a vaccine, a geographically tailored vaccine immunogen for deployment at a country level should be based on detailed information of circulating viral subtypes [8]. Therefore, the development and clinical testing of potential vaccines will require a comprehensive country-specific understanding of the distribution of the circulating subtypes.

For Belgium, a few publications concerning the circulating HCV genotypes have been published during the last decade [21–27]. However to our knowledge, only one of them, published in 2003 [21] was nationwide representative. Other available studies are regional studies [22, 23] or focused on specific risk groups such as drug users [24, 25] or specific HCV genotypes [26,27], and thus are not representative for the nation or the general population.

In this study, we describe the results from a retrospective multicenter study relating the distribution of the circulating HCV genotypes in Belgium between 2008 and 2015.

## Materials and methods

### Study design

A cross-sectional study was designed making use of the available genotyping data.

All 19 Belgium clinical laboratories performing HCV genotyping for routine analysis agreed to participate. The list of these participating laboratories was based on the list of laboratories receiving a reimbursement during the study period for their HCV genotyping analyses and was kindly provided by the national institute for health and disability insurance (RIZIV-INAMI, Brussels, Belgium). Reimbursement of the genotyping analysis is restricted only once per patient and if the start of treatment is intended. The intention to treat depends on either the fibrosis status of the infected patient or for patient groups belonging to specific risk profiles i.e. dialysis patients, pregnant patients, co-infected patients with HIV or HBV.

The participation by the clinical laboratories was voluntary and without remuneration. They collected data on a limited number of variables from all HCV genotyping analyses performed between 1/01/2008 to 31/12/2015.

The encoded variables included the genotyping and subtyping results and some patient´s demographic data allowing the identification of duplicates i.e. date of birth, gender and place of residence (postal code) [28]. In addition, the date of analysis, the laboratory method used and the genomic region of amplification to perform the genotyping analysis were recorded as well. For confidence reasons, all data transfers and further analysis were kept anonymous towards the patient.

### Data collection

All data were assembled under standardized form by the clinical laboratories and transferred to a central database located at the national institute of public health, Sciensano [28].

The requested information was limited to the data available in the laboratory information systems (LIMS) of the participating laboratories. This implied that no clinical or epidemiological data was collected i.e. co-infection, transmission routes, stage of hepatitis, acute versus chronic, date of transmission.

Thereby, the correlation of genotypes with clinical status or other epidemiological characteristics of the patients were out of the scope of this study.

Table 1 describes the different tests used by the participating laboratories to obtain the genotyping result. Those were used according to the manufacturer description. The requested laboratory results were limited to the genotype and subtype. Since the sequences were not requested, phylogenetic analyses were out of the scope of this study.

**Table 1:** Overview of the collected data obtained from the participating laboratories after deduplication of patients.

	**Number (n = 11,033)**	**Frequency (%)**
**Gender**		
**M**	6768	61.3
**F**	4247	38.5
**UNK**	18	0.2
**Year of genotyping**		
**2008**	1342	12.2
**2009**	1123	10.2
**2010**	1200	10.9
**2011**	1422	12.9
**2012**	1582	14.3
**2013**	1389	12.6
**2014**	1359	12.3
**2015**	1616	14.6
**Residence (regions and provinces)**		
**Flemisch community**	4642	42.1
**Antwerpen**	1929	17.5
**West-Vlaanderen**	993	9.0
**Vlaams-Brabant**	721	6.5
**Oost-Vlaanderen**	531	4.8
**Limburg**	468	4.2
**French speaking community**	3451	31.3
**Liège**	1318	11.9
**Hainaut**	1103	10.0
**Brabant-Wallon**	363	3.3
**Namur**	358	3.2
**Luxembourg**	309	2.8
**Brussels Capital**	2658	24.1
**UNK**	282	2.6
**Number of participating laboratories**	19	
**Age group (years)**		
**00–04**	14	0.1
**05–09**	12	0.1
**10–14**	6	0.05
**15–19**	56	0.5
**20–24**	216	2.1
**25–29**	606	5.5
**30–34**	931	8.4
**35–39**	1205	10.9
**40–44**	1459	13.2
**45–49**	1554	14.1
**50–54**	1319	12.0
**55–59**	976	8.8
**60–64**	774	7.0
**65–69**	608	5.5
**70–74**	485	4.4
**75–79**	354	3.2
**80–84**	177	1.6
**85–89**	58	0.5
**90–94**	10	0.1
**>95**	2	0.02
**UNK**	211	1.2
**Testmethod**		
**INNO-LiPA**	8484	76.9
ABBOTT*	2169	19.6
HOMESEQ*	360	3.3
O (+UNK)*	20	0.2

(*) ABBOTT: RealTime HCV Genotype II Assay (Abbott Molecular, Des Plaines, IL, USA); HOMSEQ: homebrewed method; O (+UNK): Other or unknown method.

All data were collected using an Excel (Microsoft, Redmond, USA) template using harmonized variable names with well-defined coded values and formats. Patient duplicates were identified based on the sample identification or patient demographic data i.e. date of birth, gender and postal code. Duplicates were defined as cases of identical date of birth, gender, postal code and genotype and subtype. In case of duplicates, the record without missing information or with the first date of diagnosis was maintained in the database.

### Statistical analysis

Epidemiological and graphical analyses were performed using Excel (Microsoft, Redmond, USA) or an in-house developed Epistat (https://epistat.wiv-isp.be/) module [28].

### Approval ethic committee

The study described in this manuscript was approved by the Ethic committee of the Université Catholique de Louvain (UCL), Belgium, IRB00001530, approval number 2016/16FEV/054.

It was recommended to take the necessary measures to respect the confidentiality of the medical data by anonymization of the patients. No informed consent was requested to obtain and include data from medical records.

The obtained data from HCV infected patients could not be publicly shared due to the presence of sensitive patient information. However, aggregated datasets can be graphically drawn from the in-house developed Epistat (https://epistat.wiv-isp.be/) module [28] upon request of an access. In order to be compliant with the recommendation of the ethic committee but still following the policies of the journal, a dataset with the principal variables is accessible (S1 Dataset).

## Results

### Description of collected data

A total of 12,311 records were assembled by the 19 participating laboratories performing HCV genotyping during the indicated study period from 2008 to 2015. In total 14 records were received which were classified as co-infected or which could not be classified within the genotype 1–7 i.e. 4 records with genotype 2k/1b, 6 ‘co-infections’ with genotype 1 and 4. As these were considered as a minority within the data, these were classified as ‘other’ genotypes and not further analysed.

After cleaning and deduplication of the records, based on the similarity of sample identification or patient identification, 11,033 out of 12,311 were validated for further analysis.

A higher number of genotypes were recorded for males as compared to females (M/F = 1.59).

The median yearly number of analyses was 1,374 (range 1,123–1,616).

The geographical distribution of the 19 participating laboratories (data not shown) according to the Belgian regions were 4 in the Brussels Capital, 12 in the Flemish community (north part of the country) and 3 in French speaking community (south part). Despite the higher proportion of participating laboratories from the Flemish community, the representativeness of the residence of the included patients (Table 1) reflected the density of the Belgian population in the regions i.e. respectively 42.1%, 31.3% and 24.1% for the Flemish community, the French speaking community and the Brussels Capital as compared to a population percentage of 57.5%, 32.0% and 10.5% [29].

The number of analyses was highest for the patient population between 40–49 years.

The VERSANT HCV Genotype 2.0 Assay Line Probe Assay (INNO-LiPA, Siemens Healthcare, Tarrytown, NY, USA) was the technique most in use (76.9%) during this study period.

### Overall distribution of HCV genotypes

**Fig 1:**
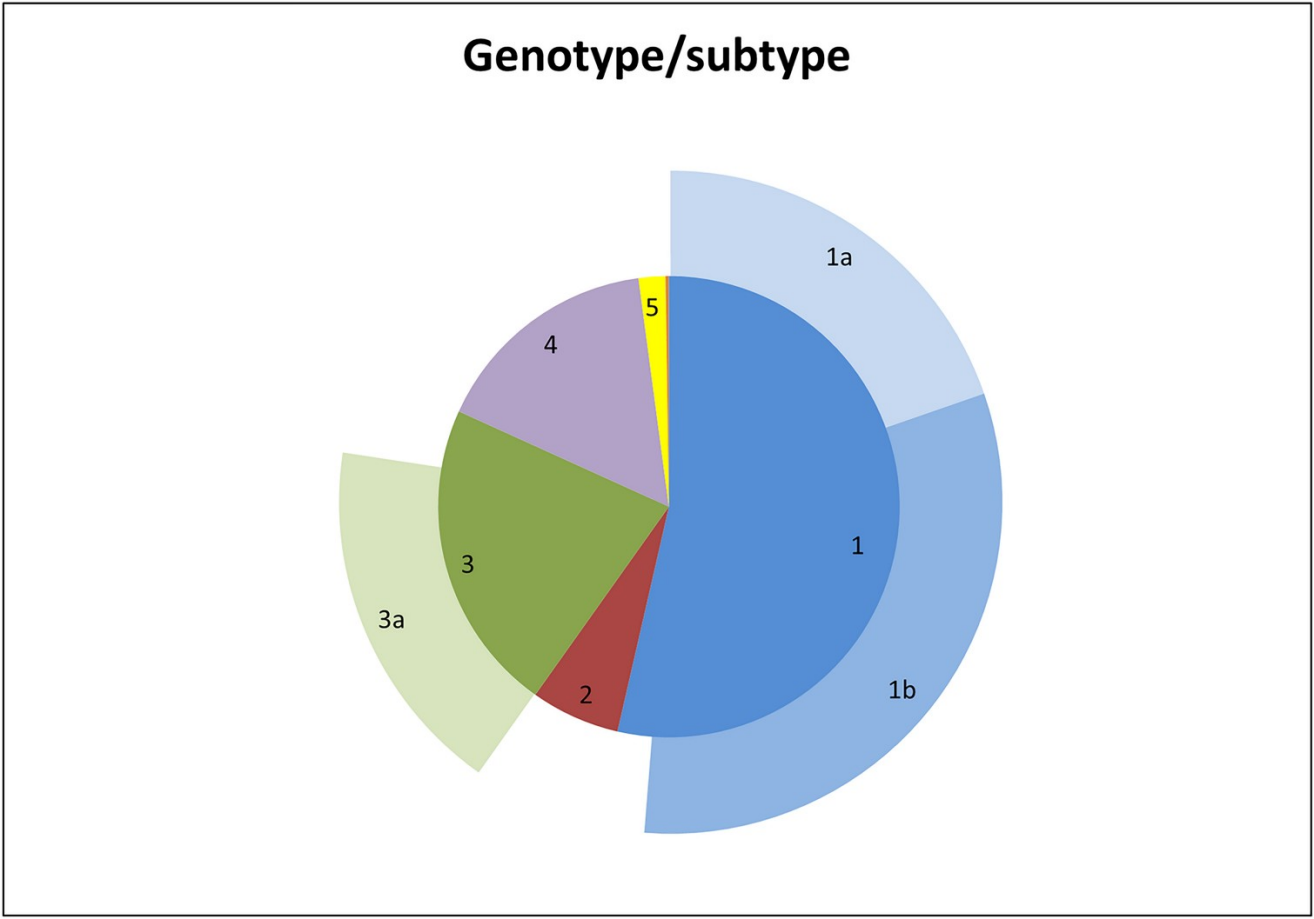
Schematic presentation of the genotype distribution (inner circle) within the Belgian population and the distribution of subtype 1a, 1b and 3a in relation to their genotype (outer circle). Due to the low prevalence, the genotype 7 cases were not shown.

The global distribution of genotypes and subtypes is schematically presented in Fig 1 and Table 2.

**Table 2:** Comparison of genotype distribution and the main study characteristics between the current study and previously published studies describing the HCV genotyping in the Belgian general population.

	**Current study**	**Verbeeck *et al*. [27]**	**Gérard *et al*. [22]**
**Participating patients (n)**	11033	2301	1726
**Study period**	2008–2015	2001–2009	1992–2002
**Region**	National	Flanders	Liège
**Questionnaire**	No	Yes	Yes
**Study protocol**	retrospective	prospective	retrospective
**method**	Multiple commercially available assays	INNO-LiPA*	INNO-LiPA*
**Gender (M/F)**	1.59	1.53	1.41
**Genotype (%)**			
**1**	53.6	60.9	61.5
**2**	6.2	6.3	11.7
**3**	22.0	20.3	14.0
**4**	16.1	8.0	11.0
**5**	1.9	4.5	1.6
**6**	0.2	0.04	0.2

INNO-LiPA*: The used version of the INNO-LiPA could not be unambiguously identified from the publications

Genotype 1 (53.6%) was the predominant genotype, followed by genotype 3 (22.0%), genotype 4 (16.1%), genotype 2 (6.2%), genotype 5 (1.9%) and finally genotype 6 and 7 with 0.2% and 0.01%, respectively. Within genotype 1, subtype 1b (31.6%) exceeded subtype 1a (19.7%), while 2.3% of genotype 1 could not be assigned to either of these subtypes. The genotyping methods used were INNO-LiPA and Abbott for respectively 67.3% and 30.6% of these non-assigned cases (data not shown).

Out of 2422 (22%) determined genotype 3, 1938 (17.6%) cases were assigned to subtype 3a.

Table 2 describes the main characteristics between the current study and previous studies performed in the Belgian general population. It demonstrates the similarities and differences of the obtained results in relation to the study protocol. Although the study of De Cock *et al*. [21] was considered as a national study, it only included a limited number of patients (n = 68). As it was published in 2003 (study period not described), we considered this study as outdated and therefore did not add the results in Table 2.

Due to the overlap in study period between the 3 different studies, the entire period ranged from 1992 till 2015. However due to differences in the study design, data cannot be combined.

The current study showed that the prevalence for genotype 2 and 3 are in line with the study from Verbeeck *et al*. [27] while for genotype 5 results are more in line with the study of Gérard *et al*. [22].

Differences in genotype distribution with previously described studies were mainly observed for genotype 1 (53.6% as compared to 60.9 and 61.5%) and genotype 4 (16.1% as compared to 8.0 and 11.0).

### Geographical distribution and trend analysis

**Fig 2:**
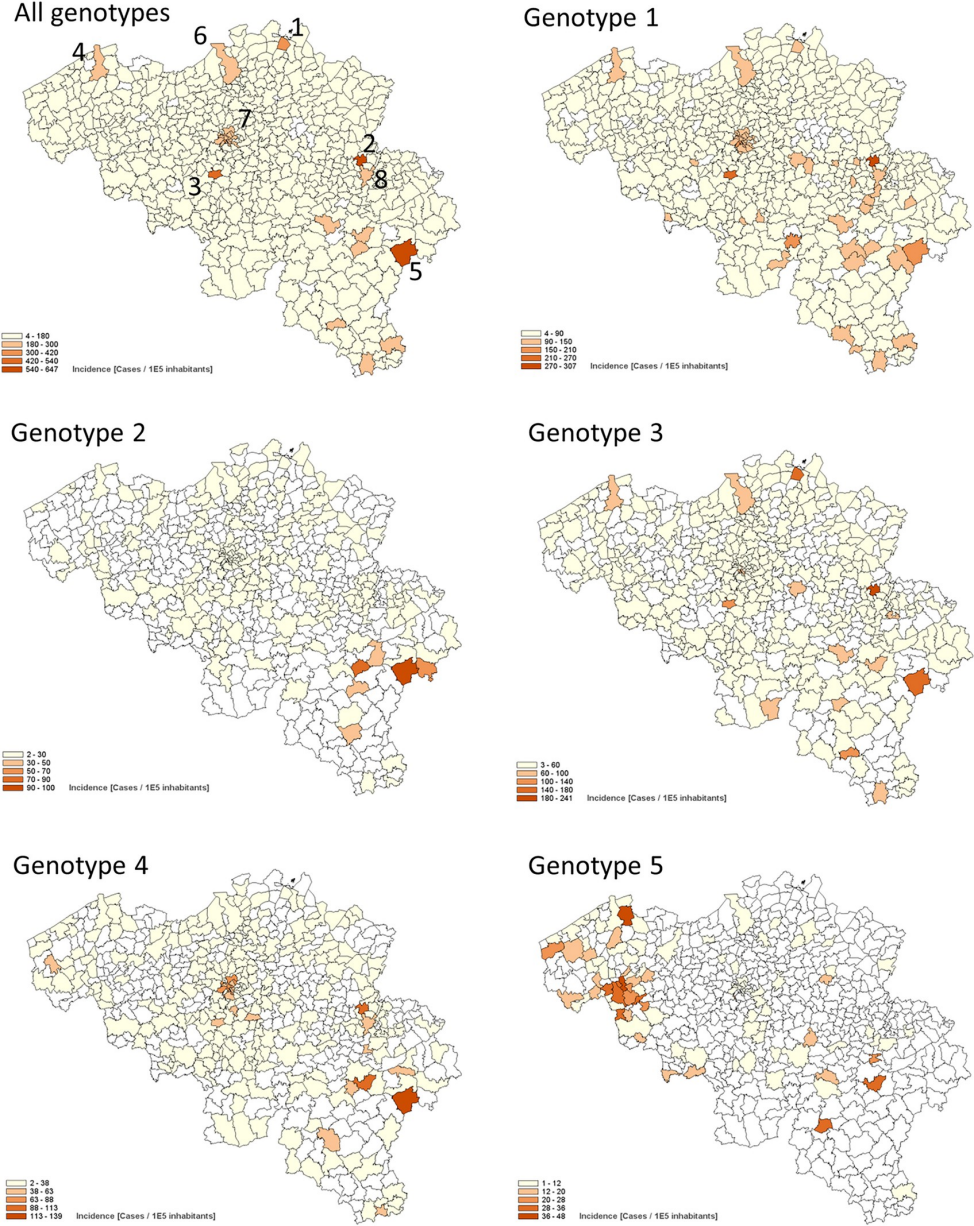
Geographic distribution at municipality level by genotype, based on the residence of the patients from 2008 to 2015. In order to visualize the differences in distribution, range categories were adapted for each genotype. Due to the low prevalence, the geographical distribution for genotype 6 cases was not shown. Numbers on the map represents locations of a few prisons i.e. Merksplas (1), Jupille (2), Ittre (3), Brugge (4), or asylum centers, Gouvy (5). As compared to metropolitan cities i.e. Antwerp (6), Brussels (7), and Liège (8).

Different patterns of genotype distribution by municipalities are shown in Fig 2.

The geographical distribution revealed no particular gradient across the country. Neither could we demonstrate a higher incidence in the metropolitan cities such as Brussels, Antwerp, Liège or Charleroi.

However higher distributions were observed in the municipalities where prisons or asylum centra are located i.e. Merksplas, Jupille, Ittre, Brugge, Gouvy. For these municipalities the distrubutions are high for genotype 1 and 3, but less for other genotypes.

Surprisingly, a cluster of patients with genotype 5 was found in West Flanders (western part of country) where the prevalence was 6 times higher than the national average. During the study period a decline in yearly frequency of this genotype 5 was observed from 3.6% (n = 48) in 2008, 2.3% (n = 26) in 2009 and 1.6% (range 1.2–2.0) during the remaining years 2010–2015 (data not shown). Of these, 120 out of 207 patients infected with genotype 5 reside in the province of West Flanders.

Despite the decline in genotype 5 and a minor one for genotype 2 from 7.3% to 5.2%, no trend in the evolution of the distribution of genotypes between 2008 and 2015 was observed.

### Distribution of genotypes in relation to gender and age

**Fig 3:**
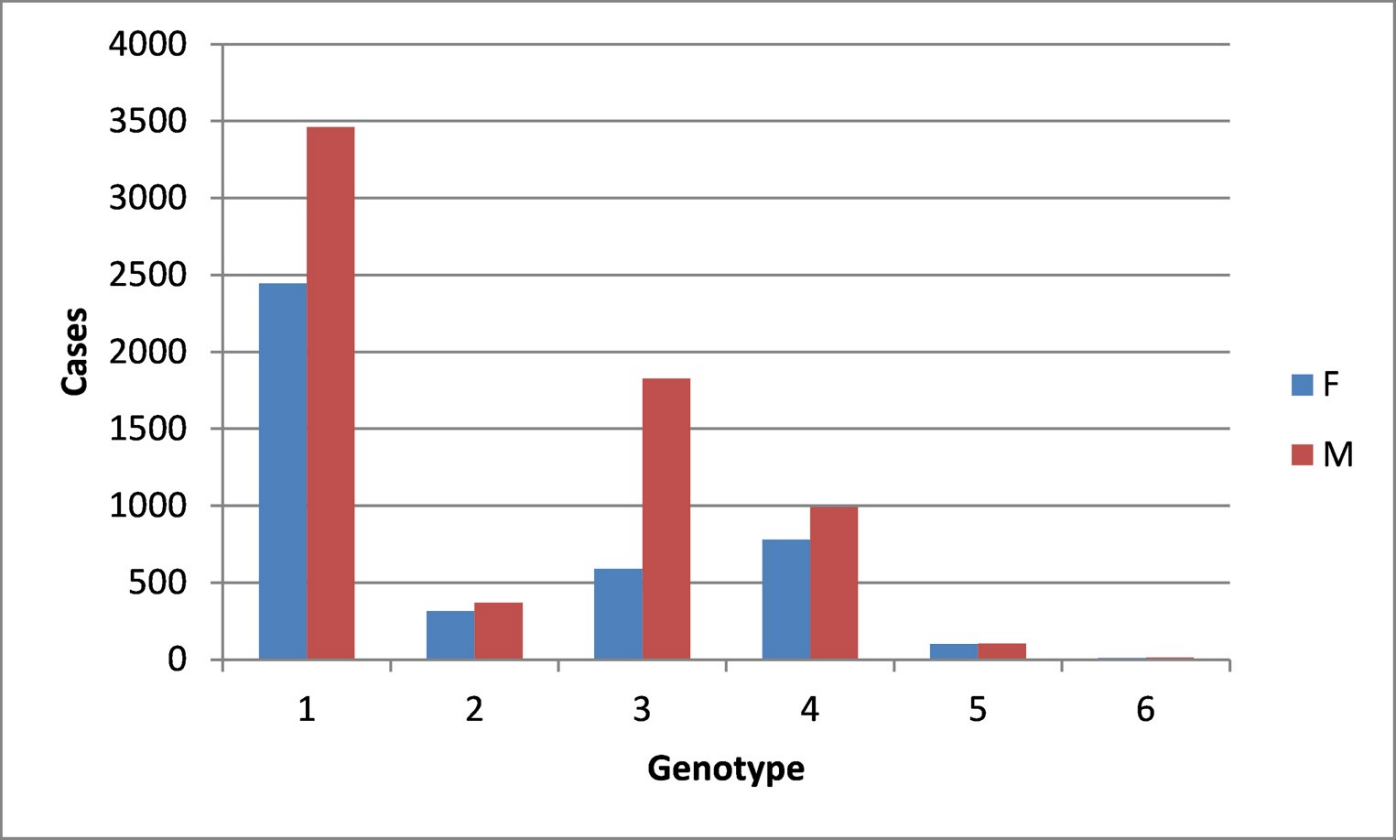
Frequency of the genotypes in function by the gender. Due to the low prevalence, the age distribution for genotype 7 cases was not shown.

All genotypes were more prevalent in males than in females (Fig 3). The overall M:F ratio was 1.59 and was mainly driven by the high M:F ratio of 3.03 for patients infected with genotype 3.

**Fig 4:**
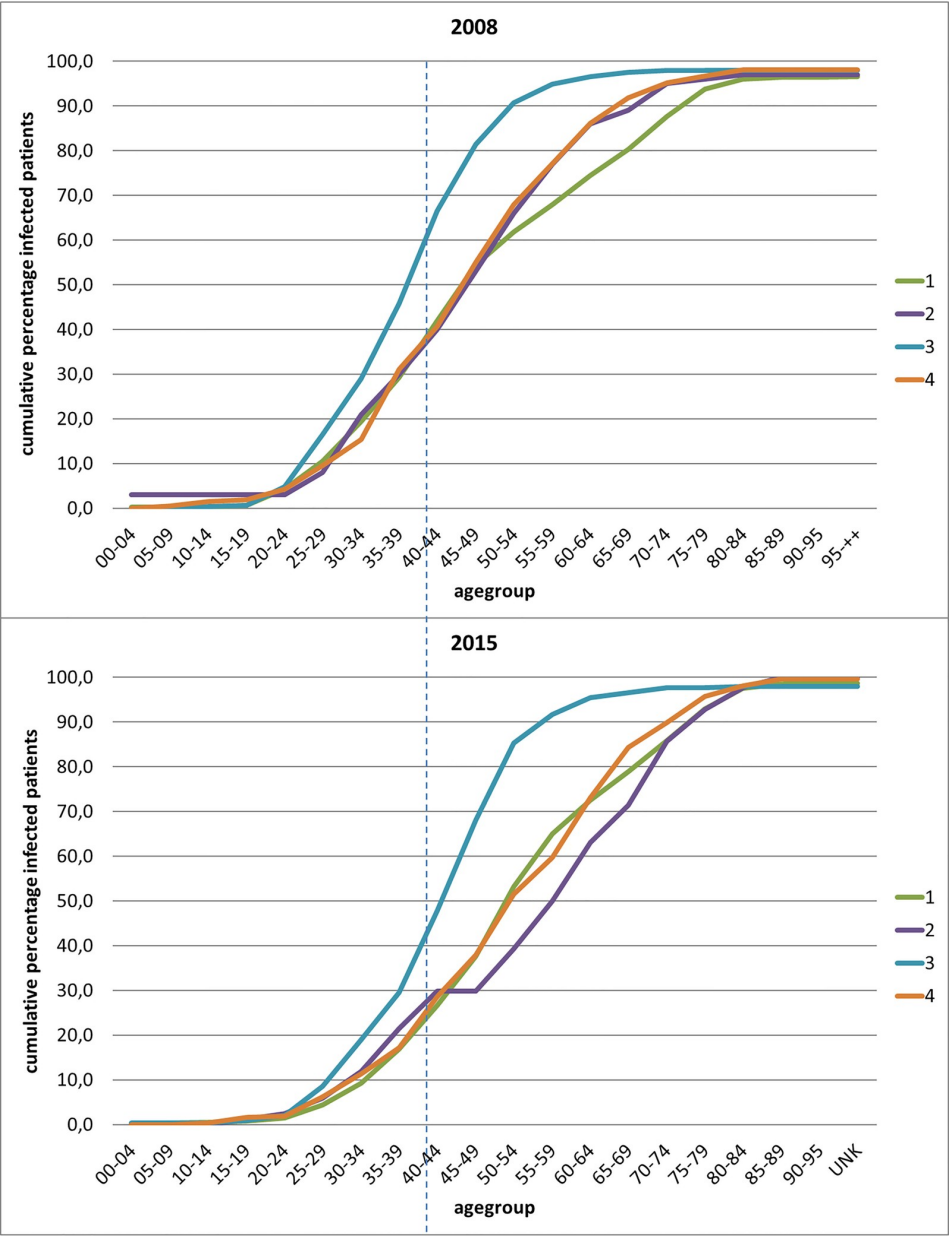
Cumulative percentage of infected patients for the different age groups and genotypes 1–4 at the time point 2015 as compared to 2008. The dashed line represents an imaginary line reaching the age of 40 years to demonstrate the shift in age groups between 2008 and 2015.

Our study found differences in the median age of the patients infected with different genotypes (Fig 4). Patients infected with genotype 3 had the lowest median age (41.7y) while those infected with genotype 5 had the highest median age (63.0y). The median age for the patients infected with all other genotypes was higher than 50.2 years of age.

The median age of patients for whom a genotyping assay was performed in 2008 is younger for all genotypes than in 2015. Fig 4 highlight the decrease in proportion of patient population of less than 40 years of age (dashed line) for whom a genotyping analysis was performed. It shows that for genotype 3 infected patients, the proportion was about 60% in 2008 and decreased to 40% in 2015. For the other genotypes it decreased from about 35% to 25%.

### Distribution of genotypes in relation to date of testing and test method

As shown in Table 1, a slight yearly increase in the number of genotyping tests was recorded between 2008 and 2015.

This study also allowed us to assess what method was the most widely used by the microbiology laboratories in Belgium to determine HCV genotypes during the study period. The most widely used method was Siemens reverse hybridization (INNO-LiPA) (76.9%), followed by Abbott real-time PCR (19.6%) and sequencing (3.3%) by homebrewed assay. During the study period the use of the INNO-LiPA method was constantly decreasing in favor of a constantly increasing use of the Abbott assay (data not shown). However, no correlation could be observed between the applied test method and the observed genotypes.

## Discussion

In the current study, all performed genotyping results from 2008 to 2015 were collected in a central database allowing the analysis of the HCV genotype distribution with national coverage. As all Belgian laboratories performing the genotyping test participated to this study, the used methodology to collect and centralize data for epidemiological analysis will be exemplary for future epidemiological studies.

The geographical representativeness is guaranteed by the participation of all laboratories performing the genotyping assay and the availability of the place of residence of patients (Table 1).

It was previously described that a high incidence of diagnosed HCV cases by qualitative or quantitative RNA confirmation tests was observed in the metropolitan municipalities such as Brussels, Antwerp, Liège or Charleroi [4]. In the current study, we collected genotyping information which might be considered as a measurement of the intention to treat and thus more downstream of the continuum of care process as compared to the diagnosis step. Based on the incidence calculations, we demonstrated that the patients for whom a genotyping was performed are rather residing in non-metropolitan areas. As demonstrated in Fig 2, increased incidences of patients for which a genotyping was performed were observed in municipalities harbouring prisons and asylum centres. This important finding is especially the case for patients infected with genotype 1 and 3.

In order to fulfil the goal of the elimination plan, as proposed by the WHO [2], it will be therefore of utmost importance to pay special attention to the high prevalence groups of prisoners and migrants from endemic regions. The Ministries of justice and migration should therefore play a crucial role in the development and implementation of the screening, prevention and treatment policies to fulfil this elimination plan.

Results from this study were compared with those from previous studies for which sufficient patients were included [22, 27]. Study periods overlapped: the study period of the one described by Gérard *et al*. [22] was 1992–2002, the one from Verbeeck *et al*. [27] was 2001–2009 and the current study 2008–2015. Despite this overlap, results could not be combined due to the differences in study protocol such as patient inclusion criteria and geographical representativeness. Therefore a trend analysis for the combined period 1992–2015 could not be performed. However, we could clearly demonstrate differences in distribution levels of genotypes between the different studies. Whether this is caused by a trend in circulating genotypes in Belgium or by the differences in study protocol or geographically representation of the patient population, could not be unravelled.

The study of Gérard *et al*. [22], conducted by 5 hospitals from the province of Liège was continued till 2007 and described in a poster presentation by Loly *et al*. [23] with limited data at the level of genotype trends. However the circulating genotypes observed in this regional study of Loly *et al*. [23] with a limited number of patients in 2007 (n = 158) are in line with the one observed in this study for 2008. Although a significant evolution of HCV genotypes for 2884 patients between 1992 and 2007 was demonstrated for genotype 1a (3% to 11%), 1b (66% to 36%), 3 (10% to 21%) and 4 (5% to 16%), no such evolution was observed in the current study.

As described by Esteban *et al*. [30], the epidemic of HCV infection is continuously evolving due to an improvement of blood transfusion safety, improvement of healthcare conditions, increased immigration from endemic regions and intravenous drug use safety programs. Furthermore, an increasing incidence of HCV has been observed among HIV-positive men who have sex with men [31].

A change in risk profile was demonstrated by Loly *et al* [23] during the study period from 1992 to 2007 conducted in the region of Liège. It is tempting to hypothesize that the change in risk profile might have an impact on the evolution of circulating genotypes. In the current study, only a limited change in distribution of genotypes was observed. As no clinical or epidemiological data were collected in the current study, we could not demonstrate whether a stabilization in risk factors could explain the lack of evolution in genotype distribution. Nevertheless, after 25 years of implementation of blood transfusion safety programs and improved prevention programs in healthcare, it is generally agreed upon that intravenous drug use now is the main risk factor for HCV transmission [30].

A shift in the median age of the patient population for which a genotyping result was performed was observed for all genotypes. In general, patients infected with genotype 3 were much younger as compared to patients infected with other genotypes. These differences in age distribution between genotype 3 and the other genotypes and whether this is linked to their increased prevalence within the intravenous drug users necessitate further studies to investigate the cause.

We demonstrated an increased presence of genotype 5 in the province West Flanders (Fig 2). Verbeeck *et al*. [27] described the presence of a genotype 5a cluster in West Flanders between 2001 and 2009. The inclusion of the patient population from limited provinces, including the province West Flanders where this cluster was observed, caused probably the high prevalence of this genotype in their study (Table 2). As the province of West Flanders is located at the border of France, we investigated but could not confirm whether a similar increased presence was also measured in the departments of “France Nord et Pas-de-Calais”; the department at the Belgian border.

The results from this study, conducted in the general population, allowed us to compare the HCV genotype distribution with subpopulations such as the one among drug users.

Matheï *et al*. [24] and Micalessi *et al*. [25] described the HCV genotype distribution respectively in 152 infected drug users in 1999–2000 in Flanders and 98 infected drug users in 2004–2005 in Belgium: genotype 1 (48.7%-38%) and genotype 3 (41.2%-49%) were predominant, followed by genotype 4 (8.8%-9%), and genotype 2 (1.4%-2%).

Comparing these results with the current study demonstrates an increased presence of genotype 3 among drug users as compared to the general population in Belgium. On the other hand, genotypes 1 and 2 were relatively less prevalent in the drug user subpopulation. For genotype 4, the prevalence is similar to the prevalence in the general population during the same study period as described in previous reports [22, 27]. The availability of information concerning the inclusion criteria of the target populations is of utmost importance for the interpretation and final conclusions of prevalence studies.

### Limitations of the study

Although all genotyping test performed between 2008 and 2015 for which a reimbursement of the test was performed were included in the analysis, the sampling strategy should be considered as a convenience sample. Since we are not sure whether all HCV infected patients underwent a HCV genotyping assay, we cannot consider this study as a true population based study. Sampling is therefore subject to all of the biases involved upstream of the genotyping assay. Since no limitations were given to specific high prevalence groups such as persons who inject drugs, prisoners, migrants from endemic regions, this study focused on the general population with residence in Belgium.

As the samples are restricted to patients being tested with the intention to treat, and not for all diagnosed HCV patients, the obtained results cannot be considered as prevalences of the HCV genotypes.

The laboratories performing HCV genotyping assays and providing the data for this study underwent quality assessments at regular frequency, demonstrating the genotyping classification with sufficient quality. Nevertheless, we were not able to measure the misclassifications of the genotypes caused by either the limitations of the assays (e.g. genotypes 3 and 6 or recombinant forms 2k/1b) or human misinterpretations.

Since the current study has a national coverage, it can be used as a baseline for further follow-up during the coming era of access to HCV direct-acting antivirals, in case of outbreak investigations or to study the impact of immigration flows from endemic regions. Since the laboratories performing the genotyping tests are known, the methodology to collect the data described, and analysis programs available, the study can be prolonged at regular frequencies. Seen the success of participation for the HCV genotyping study, we could think of enlarging it to other infectious diseases and thereby opening new epidemiologic perspectives.

## Conclusions

This study describes the most recent data on HCV genotype distribution in the Belgium HCV infected patients with a national coverage. It allowed a clear and objective view of the circulating HCV genotypes in the general population in Belgium. Knowing the actual therapeutic success rates, it will help health authorities in the establishment of cost effectiveness determinations before implementation and reimbursement of new treatment strategies.

In the future, this baseline characterization of the circulating genotypes will be indispensable for continuous surveillance, especially for tracking of genotypes seen in HCV outbreaks within local high-risk groups, or unusual genotypes transmitted by immigrants, expats, or travelers arriving from endemic regions.

## Supporting information

S1 Dataset Dataset of the HCV genotype records containing the principal variables and collected by the participating laboratories. (XLSX)
